# Extracts from *Valsonectria inflata*, a Soil-Derived Fungus, Inhibit Human Coronavirus OC43 Replication

**DOI:** 10.3390/ijms27146328

**Published:** 2026-07-16

**Authors:** Chunghyeon Lee, Siyun Lee, Seungju Cho, Sumin Kim, SeonJu Park, Mi Hyeon Cho, Eunji Cho, Jayhyun Park, Hyung-Gwan Lee, Hye Yeon Mun, Chang Soo Lee, Junsoo Park

**Affiliations:** 1 Division of Biological Science and Technology, Yonsei University, Wonju 26493, Republic of Korea; 2 Metropolitan Seoul Center, Korea Basic Science Institute (KBSI), Seoul 02841, Republic of Korea; 3 Analysis & Evaluation Department, Korea Mine Rehabilitation and Mineral Resources Corporation, Wonju 26464, Republic of Korea; 4 R&D Team, Korea Mine Rehabilitation and Mineral Resources Corporation, Wonju 26464, Republic of Korea; 5 Cell Factory Research Center, Korea Research Institute of Bioscience and Biotechnology (KRIBB), Daejeon 34141, Republic of Korea; 6 Biological Resources Research Department, Nakdonggang National Institute of Biological Resources, Sangju 37242, Republic of Korea

**Keywords:** human coronavirus, *Valsonectria inflata*, *Acremonium inflatum*, antiviral, fungus

## Abstract

Coronaviruses are responsible for both severe diseases, such as COVID-19, and mild illnesses, such as the common cold. Because coronaviruses are expected to remain continuously prevalent, alternative therapeutic strategies against coronavirus infections should be developed to respond to newly emerging variants. Since many antibiotics, including penicillin, have been isolated from fungi, we screened fungal extracts for antiviral activity against human coronavirus and found that the extract of *Valsonectria inflata* (VIE) exhibited antiviral effects against human coronavirus. Western blot analysis showed that VIE treatment decreased coronavirus protein expression. Quantitative RT-PCR (qRT-PCR) and plaque formation assays demonstrated that VIE treatment reduced coronavirus production, while scanning electron microscopy (SEM) analysis further confirmed a decrease in the production of infectious viral particles. Finally, VIE treatment ameliorated coronavirus-induced cytotoxicity. We also analyzed the components of VIE, and high-resolution electrospray ionization mass spectrometry (HR-ESI-MS) revealed that VIE contains various secondary metabolites, including terpenoids. These results suggest that VIE may serve as a potential antiviral agent against coronavirus infections.

## 1. Introduction

Fungi have provided many valuable pharmaceutical compounds, including antibiotics, for human use [1,2]. Penicillin, a representative antibiotic, and cephalosporin were isolated from *Penicillium notatum* and *Cephalosporium acremonium* (formerly *P. cephalosporium*), respectively, while fusidic acid, an antibiotic effective against Gram-positive bacteria, was isolated from *Fusidium coccineum* [3,4,5]. Griseofulvin and echinocandin B are antimycotic agents derived from *P. griseofulvum* and *Aspergillus nidulans*, respectively [6,7]. Moreover, cyclosporin, an immunosuppressive drug, and taxol, a widely used anticancer drug, were isolated from *Tolypocladium inflatum* and *Taxomyces andreanae*, respectively [8,9]. Because the number of fungal species is estimated to reach several million, while less than 10% of these species have been identified, fungi are expected to contain many additional valuable pharmaceutical compounds for human medicine [10,11].

Recently, the emergence of SARS-CoV-2, a bat-derived coronavirus, has severely affected nearly every aspect of human life; however, coronaviruses have long been responsible for a significant proportion of common cold cases [12,13]. Human coronavirus strains, including HCoV-OC43, HCoV-229E, HCoV-NL63, and HCoV-HKU1, have circulated widely in humans and are responsible for approximately 20–30% of common cold cases [14,15]. HCoV-OC43 is an enveloped, positive-sense single-stranded RNA virus and a common cause of mild upper respiratory tract infections [16]. HCoV-OC43 belongs to the Betacoronavirus genus, which also includes SARS-CoV-2; therefore, HCoV-OC43 is widely used as a biosafety level 2 model for studying coronavirus replication and evaluating antiviral agents [17]. Although HCoV-OC43 infections are generally mild and self-limiting, effective antiviral therapies specifically targeting this virus are limited [18]. Therefore, the identification of novel antiviral substances against HCoV-OC43 remains important, and natural products may provide promising sources of antiviral compounds.

Here, we screened fungal strains for antiviral activity and identified *Valsonectria inflata* as a promising candidate with inhibitory effects against human coronavirus. *V. inflata* is a soil-derived fungus that was formerly classified as *Acremonium inflatum* before being reclassified as *V. inflata* in 2023 [19]. Among the fungal extracts evaluated in the functional screening, the *V. inflata* extract exhibited the strongest inhibitory effect on HCoV-OC43 protein expression. Although several other fungal extracts also reduced viral protein levels, some caused substantial host–cell toxicity under the screening conditions. In contrast, the *V. inflata* extract showed marked antiviral activity without a corresponding reduction in host–cell viability. Moreover, no pharmacological or antiviral activity of *V. inflata* has previously been reported. Therefore, *V. inflata* was selected for further investigation based on its antiviral potency, favorable selectivity, and pharmacological novelty. To the best of our knowledge, this is the first study to demonstrate the pharmacological activity of a *V. inflata* extract. Treatment with the *V. inflata* extract reduced coronavirus RNA and protein levels and decreased the production of infectious viral particles. These findings suggest that *V. inflata* may represent a promising natural source of antiviral compounds for the development of therapeutic agents against coronavirus infections.

## 2. Results

### 2.1. Valsonectria inflata Extract (VIE) Decreased Coronavirus Protein Expression

**Figure 1:**
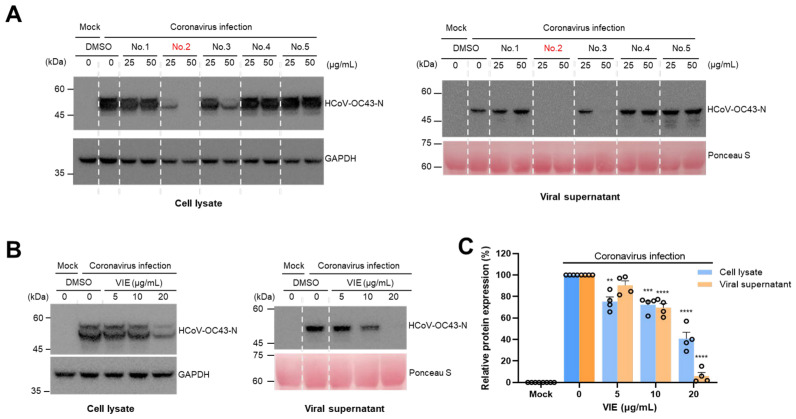
*Valsonectria inflata* extract (VIE) effectively reduces coronavirus replication. (**A**) Treatment with *Valsonectria inflata* extract (VIE) reduced coronavirus protein levels in virus-infected cells (left panel) and in the viral supernatant (right panel). RD cells were infected with HCoV-OC43 and co-treated with a series of candidate extracts for 72 h. Western blot screening revealed that VIE (No. 2) markedly decreased coronavirus protein expression. An anti-HCoV-OC43 antibody was used to detect the nucleocapsid protein of HCoV-OC43, and the detected signal was labeled as ‘HCoV-OC43-N’ in the figures. Viral supernatant refers to the culture medium collected from infected cells, and Ponceau S staining was used as a loading control for the viral supernatant. (**B**) VIE treatment inhibited coronavirus protein expression in a dose-dependent manner. RD cells were infected with HCoV-OC43 and treated with the indicated concentrations of VIE. Cell lysates and the viral supernatant were collected for Western blot analysis. (**C**) Quantification of relative coronavirus protein expression levels. Error bars represent the standard error of the mean (SEM; N = 4). For comparisons among multiple groups, one-way ANOVA followed by Dunnett’s post hoc test was used to compare each treatment group with the virus-infected control group. A *p*-value of < 0.05 was considered statistically significant., ** *p* < 0.01, *** *p* < 0.001, **** *p* < 0.0001.

To identify antiviral compounds from fungal extracts, we screened several fungal extracts using human coronavirus (HCoV-OC43) infection models and found that treatment with *Valsonectria inflata* extract (VIE) reduced the expression of coronavirus proteins, suggesting that VIE possesses antiviral activity against coronavirus (Figure 1A). RD cells were infected with HCoV-OC43 and incubated with various fungal extracts. The cell lysates were subjected to Western blot analysis using an anti-HCoV-OC43 antibody. An anti-HCoV-OC43 antibody detects the nucleocapsid protein (N) of HCoV-OC43 [20]. The fungal strains used for screening were obtained from the Freshwater Bioresources Culture Collection (FBCC), Republic of Korea. We selected fungal extracts that markedly reduced viral protein expression in the Western blot screening. During the course of the experiments, we performed fungal characterization to confirm that the isolate was *Valsonectria inflata* (Supplementary Figure S1). Most fungal extracts did not affect coronavirus protein (HCoV-OC43-N) expression; however, VIE treatment markedly decreased the expression of coronavirus proteins (Figure 1A). To further confirm these findings, cells were treated with different concentrations of VIE (0, 5, 10, and 20 μg/mL), and VIE treatment reduced coronavirus protein expression in a dose-dependent manner (Figure 1B). In addition, VIE treatment significantly decreased coronavirus protein levels in both the viral supernatant and cell lysates (Figure 1C). HCoV-OC43-N protein expression was normalized to GAPDH expression and Ponceau S staining (Figure 1C). We calculated the half-maximal inhibitory concentration (IC_50_) values, which were 16.94 μg/mL (R^2^ = 0.8215; 95% CI, 14.58–20.05 μg/mL) for intracellular viral proteins and 12.33 μg/mL (R^2^ = 0.9751; 95% CI, 11.39–13.33 μg/mL) for viral proteins in the viral supernatant. Because coronavirus proteins detected in the viral supernatant originate from virus particles released from infected cells, these results suggest that VIE inhibits coronavirus replication and viral particle production.

### 2.2. VIE Treatment Decreased Coronavirus-Induced Cytotoxicity

**Figure 2:**
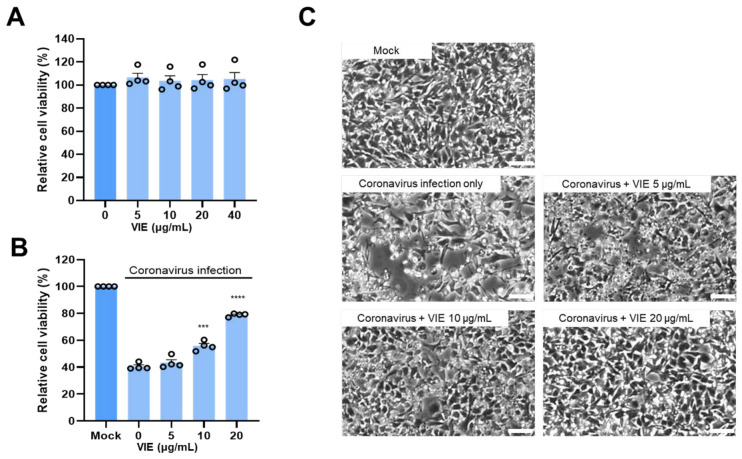
VIE attenuates coronavirus-induced cytotoxicity. (**A**) Effects of VIE treatment on RD cell viability. RD cells were treated with increasing concentrations of VIE, and cell viability was assessed using the MTT assay. (**B**) VIE treatment alleviated virus-induced cell death in a dose-dependent manner. Error bars represent the standard error of the mean (SEM; N = 4). Statistical differences across groups were assessed by one-way ANOVA with Dunnett’s post hoc test, using the virus-infected group as the reference control. A *p*-value of <0.05 was considered statistically significant.*** *p* < 0.001, **** *p* < 0.0001. (**C**) Representative microscopy images of RD cells infected with HCoV-OC43 and treated with the indicated concentrations of VIE. Scale bars, 10 μm.

Previous studies have shown that coronavirus infection induces cytotoxicity, and antiviral compounds can ameliorate coronavirus-induced cellular damage [21]. Because VIE treatment reduced coronavirus protein expression, we next examined whether VIE could also alleviate coronavirus-induced cytotoxicity. First, cell viability was evaluated using an MTT assay to assess the cytotoxicity of VIE at various concentrations. Rhabdomyosarcoma (RD) cells were treated with the indicated concentrations of VIE. No significant cytotoxicity was observed at concentrations up to 40 μg/mL, and cell viability was not significantly affected by VIE treatment (Figure 2A). Next, we investigated whether VIE could reduce coronavirus-induced cytotoxicity. RD cells were infected with human coronavirus (HCoV-OC43) and treated with various concentrations of VIE (0, 5, 10, and 20 μg/mL). Virus infection alone markedly reduced cell viability to approximately 40%, indicating strong cytopathic effects (Figure 2B). However, VIE treatment restored cell viability in a dose-dependent manner, reaching approximately 80% viability at 20 μg/mL. We further examined the effects of VIE using microscopy. Coronavirus-infected cells exhibited severe cytopathic effects, including cell detachment, empty areas on the culture surface and cell rounding; however, treatment with VIE progressively alleviated these morphological changes, with substantial recovery observed at 20 μg/mL (Figure 2C). Taken together, these results indicate that VIE itself does not exhibit significant cytotoxicity and effectively alleviates coronavirus-induced cytotoxicity in a dose-dependent manner.

### 2.3. VIE Treatment Decreased Coronavirus Genome Replication

**Figure 3:**
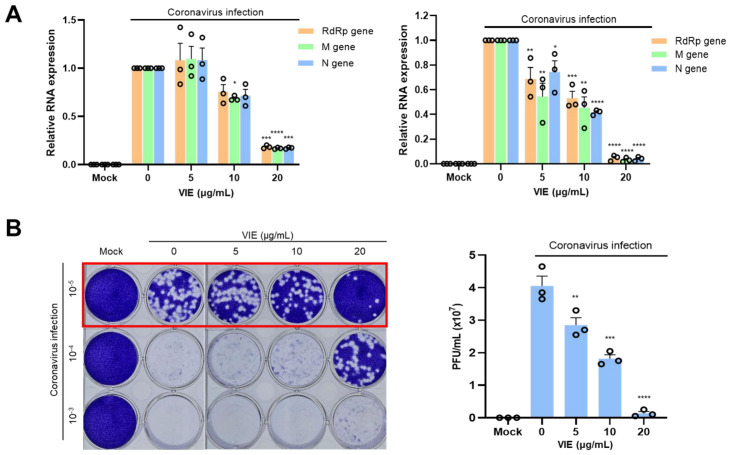
VIE attenuates coronavirus replication and viral infectivity. (**A**) VIE treatment reduced viral RNA levels in coronavirus-infected cells (left panel) and in the viral supernatant (right panel). Quantitative real-time PCR (qRT-PCR) was performed to evaluate the antiviral effects of VIE at the RNA level. Relative viral RNA abundance was normalized to RPL4 gene expression. Error bars represent the standard error of the mean (SEM; N = 3). Comparisons were performed using one-way ANOVA followed by Dunnett’s post hoc test between the infected control and VIE-treated groups. * *p* < 0.05, ** *p* < 0.01, *** *p* < 0.001. (**B**) VIE treatment impaired coronavirus propagation. Plaque assays demonstrated that viral infectivity was reduced by VIE treatment. Viral supernatant collected from coronavirus-infected cells treated with VIE was filtered and used to infect fresh RD cells for plaque assays. The number of plaques in the groups highlighted by the red box was quantified and is shown in the graph. Error bars represent the standard error of the mean (SEM; N= 3). Group-wise statistical significance was evaluated using one-way ANOVA followed by Dunnett’s post hoc test, with the virus-infected group set as the reference control. Differences were considered statistically significant at *p* < 0.05. * *p* < 0.05, ** *p* < 0.01, *** *p* < 0.001, **** *p* < 0.0001.

Coronaviruses are RNA viruses, and the level of viral RNA reflects the extent of coronavirus replication. To evaluate viral replication, RNA was isolated from infected cells (cell lysates) and the viral supernatant, followed by quantitative RT-PCR analysis. Coronavirus membrane (M), nucleocapsid (N), and RNA-dependent RNA polymerase (RdRp) genes were used as targets for quantification. Similar to the reduction observed in coronavirus protein expression, VIE treatment decreased the levels of coronavirus RNA in both cell lysates and the viral supernatant in a dose-dependent manner (Figure 3A). The IC_50_ values of VIE were calculated to be 12.67–13.55 μg/mL for intracellular viral RNA and 6.47–8.67 μg/mL for viral RNA in the viral supernatant (Table 1). Because VIE treatment reduced coronavirus RNA levels in the viral supernatant, these results suggest that VIE suppresses the production and release of coronavirus particles. To further evaluate the antiviral activity of VIE, plaque formation assays were performed using the viral supernatant collected from VIE-treated infected cells. Representative images of stained cell monolayers are shown in Figure 3B. Mock-treated cells exhibited intact monolayers without plaque formation, whereas coronavirus-infected cells showed extensive plaque formation, indicating high titers of infectious coronavirus particles (Figure 3B). Treatment with VIE markedly reduced plaque numbers in a dose-dependent manner. At 5 and 10 μg/mL, plaque formation was partially reduced, whereas at 20 μg/mL, plaques were almost completely abolished, indicating strong antiviral activity (Figure 3B). Overall, these results demonstrate that VIE treatment interferes with coronavirus replication and reduces the production of infectious viral particles.

### 2.4. VIE Treatment Decreased the Production of Coronavirus Particles in Cells

**Figure 4:**
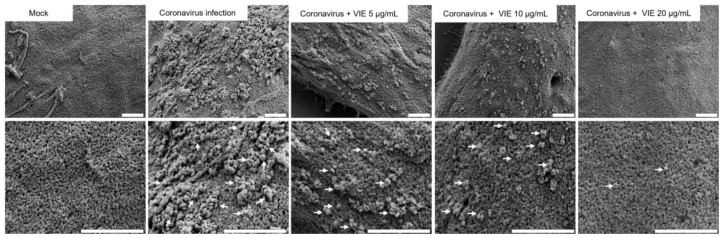
VIE disrupts the accumulation of coronavirus particles on the surface of virus-infected cells. RD cells were infected with HCoV-OC43 and treated with the indicated concentration of VIE. Representative scanning electron microscopy (SEM) images of coronavirus-infected RD cells are shown. Two different magnifications were used to visualize the overall pattern of viral particles (20,000×, upper panel) and individual coronavirus particles (50,000×, lower panel). Arrowheads indicate coronavirus particles. Scale bars, 1 μm.

Because VIE exhibited antiviral effects against coronavirus based on both protein and RNA analyses, we further examined its antiviral activity using scanning electron microscopy (SEM). Representative SEM images of cells treated with VIE are shown in Figure 4. Mock-treated cells exhibited smooth and intact surface morphology without visible viral particles. In contrast, cells infected with HCoV-OC43 were densely covered with coronavirus particles, indicating active viral replication and viral particle production (Figure 4). Compared with untreated infected cells, VIE-treated cells showed fewer coronavirus particles on the cell surface (Figure 4, upper panel). At higher magnification, individual coronavirus particles were observed in HCoV-OC43-infected cells, whereas fewer visible particles were observed after VIE treatment (Figure 4, lower panel).

### 2.5. QTOF-MS Analysis of VIE

**Figure 5:**
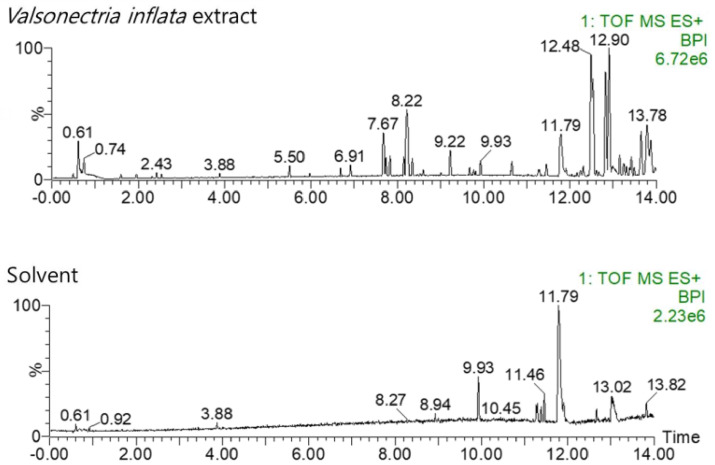
Base peak intensity chromatograms (BPI; TOF-MS ES+) of *V. inflata* extract and the solvent blank. *V. inflata* extract exhibited multiple distinct chromatographic peaks compared with the solvent blank, indicating the presence of diverse secondary metabolites.

After confirming the antiviral activity of VIE against human coronavirus, we further investigated its metabolite composition. Metabolite profiling by QTOF-MS analysis revealed that VIE contains diverse secondary metabolites (Figure 5). Several compounds were putatively annotated, whereas others were tentatively classified as terpenoid and diacylglycerol derivatives based on their accurate mass and MS/MS fragmentation patterns (Table 2). The metabolites detected in VIE included N-lauryldiethanolamine (retention time [RT] 7.67 min), tris(2,2-dimethylpropionylamino)benzene (RT 8.22 min), sphinganine (d18:0) (RT 9.22 min), a dimeric meroterpenoid derivative (RT 12.78 min), triterpenoid derivatives (RT 12.82 and 12.90 min), and diacylglycerol derivatives (RT 13.78 and 13.87 min).

## 3. Discussion

Here, we report that treatment with *Valsonectria inflata* extract (VIE) interferes with coronavirus replication and reduces the production of infectious coronavirus particles. In addition, we demonstrated that VIE treatment ameliorates coronavirus-induced cytopathic effects. These findings suggest that VIE may serve as a potential therapeutic candidate for coronavirus-related diseases. Fungi are rich sources of secondary metabolites, and many clinically important medicines have been derived from fungi [1,2]. Antibiotics such as penicillin and cephalosporin were originally discovered from fungal species, and we hypothesized that fungal extracts may also contain antiviral compounds. Therefore, we screened various fungal extracts for antiviral activity and found that *V. inflata* extract exhibited potent antiviral effects against coronavirus.

During the COVID-19 pandemic, numerous studies investigated plant-derived antiviral compounds. In contrast, fungi have not been extensively explored as sources of antiviral agents. Nevertheless, several studies support the antiviral potential of fungal extracts and metabolites. For example, methanol extracts of *Ganoderma lucidum* have been reported to inhibit both HIV-1 replication and HIV-1 protease activity [26,27]. Fungal-derived metabolites have also shown activity against HCoV-OC43. For example, sarcodonol D, a cyathane diterpenoid isolated from Sarcodon imbricatus, suppressed HCoV-OC43 infection and reduced virus-induced cellular damage, while mycophenolic acid, another fungal secondary metabolite, inhibited HCoV-OC43 replication [28,29]. Screening studies have also identified several fungal extracts with anti-HCoV-OC43 activity [30]. In addition, our previous study demonstrated that P. compactum produces antiviral compounds [21]. Collectively, these findings suggest that fungi, which are well-established sources of bioactive secondary metabolites, may represent valuable resources for antiviral drug discovery. Further screening of fungal extracts and the isolation and characterization of their active constituents are therefore warranted. *V. inflata* is a soil-derived fungus that was previously classified as *A. inflatum*, and its biological and pharmacological properties remain poorly characterized [19]. To the best of our knowledge, this is the first study to demonstrate the pharmacological activity of *V. inflata*.

Chemical profiling of the extract revealed the presence of several metabolite classes, including alkaloids, sphinganines, diacylglycerols, and triterpenoid derivatives; however, the specific bioactive compounds responsible for the antiviral activity have not yet been fully identified. Because chemical profiling studies of *V. inflata* are currently limited, a comprehensive chemical library for this fungus is not available. Therefore, further studies, including compound purification, structural elucidation by NMR spectroscopy, and complementary MS/MS analyses, will be required to identify the active compounds present in VIE. Another limitation of the metabolomic analysis is that an uninoculated PDB blank was not included as a parallel negative control. Therefore, the possibility that some detected signals originated from media-derived background components cannot be completely ruled out. Accordingly, the metabolite annotations in this study should be interpreted with caution and regarded as tentative unless supported by sufficient MS/MS fragmentation data and/or reference information. Future studies including appropriate media blank controls will be required to more accurately distinguish fungal metabolites from media-derived background signals.

Our component analysis indicated that VIE contains many metabolites (Table 2). Among these metabolites, terpenoid derivatives, including dimeric meroterpenoid and triterpenoid derivatives, are of particular interest because terpenoids have been reported to exhibit antiviral activity against coronaviruses, including SARS-CoV and SARS-CoV-2 [31,32,33]. Based on these findings, we speculate that triterpenoid derivatives in VIE may contribute, at least in part, to its antiviral activity. However, further chemical characterization and compound-specific functional validation will be necessary to identify the specific antiviral components responsible for the observed effects of VIE.

In this manuscript, we used the HCoV-OC43 strain to examine antiviral activity. Because HCoV-OC43 belongs to the genus Betacoronavirus, which also includes SARS-CoV-2, it can serve as a useful surrogate model for studying betacoronavirus replication. In addition, HCoV-OC43 is one of the human coronaviruses responsible for the common cold. Therefore, further validation using SARS-CoV-2 and other common-cold human coronaviruses would be beneficial for extending the applicability of the current findings. Moreover, although we demonstrated that HCoV-OC43 replication was downregulated by VIE treatment, the exact molecular mechanism remains unknown. In this study, we screened fungal species and identified *V. inflata* as a fungus with inhibitory activity against coronavirus replication. Therefore, further mechanistic and validation studies using additional coronavirus strains will be necessary to evaluate the broader antiviral potential of *V. inflata*. Although the IC_50_ values of VIE were calculated from dose–response curves, these values should be interpreted with caution because they were estimated using a relatively narrow concentration range and a limited number of data points. Therefore, the IC_50_ values presented in this study should be considered approximate estimates of antiviral activity rather than definitive pharmacological parameters. Further studies using a broader concentration range and additional data points will be required to determine the antiviral potency of VIE more accurately.

Another limitation of this study is the absence of a well-established positive antiviral control. Although the IC_50_ values of VIE were estimated from dose–response curves, these values do not replace the need for a reference antiviral compound to validate the experimental system and compare antiviral potency. Therefore, future studies should include an appropriate positive control to more rigorously evaluate the antiviral activity of VIE.

## 4. Materials and Methods

### 4.1. Strain Isolation, Identification, and Cultivation

The fungal strain was isolated from sediment collected from Miho-cheon in Sejong-si, Republic of Korea. The sediment sample was serially diluted to 10^2^, 10^3^, and 10^4^, and 200 µL of each dilution was spread onto potato dextrose agar (PDA) medium, followed by incubation at 15 °C for fungal isolation. The isolated fungi were preserved in 15% glycerol and stored at −80 °C.

To identify the isolated strains, mycelia cultured on PDA were harvested and transferred to tubes containing glass beads, followed by homogenization. DNA was then extracted using the NucleoSpin Plant II DNA Extraction Kit (Macherey-Nagel, Düren, Germany). The extracted DNA was used to amplify the internal transcribed spacer (ITS) region by PCR using the primers ITS1 (5′-TCCGTAGGTGAACCTGCGG-3′) and ITS4 (5′-TCCTCCGCTTATTGATATGC-3′). DNA sequence alignment, editing, and phylogenetic tree construction were performed using MEGA 12.1.2 [34]. The strains were observed using an Eclipse Ni-U microscope (Nikon, Tokyo, Japan).

For cultivation, cultures were incubated on PDA at 25 °C for 7 days in an incubator (VS-8480SR, Vision Scientific, Daejeon, Republic of Korea). Three agar plugs, each 8.75 mm in diameter, were excised from the PDA plate using a No. 4 cork borer, and each plug was aseptically cut into eight equal fragments, yielding a total of 24 agar pieces. These fragments were inoculated into 80 mL of potato dextrose broth (PDB; Difco, BD, Franklin Lakes, NJ, USA) in a 250 mL baffled Erlenmeyer flask and incubated at 25 °C with shaking at 150 rpm for 10 days in a shaking incubator (VS-8480SR, Vision Scientific, Daejeon, Republic of Korea).

### 4.2. Preparation of V. inflata Extract

After cultivation, 50 mL of the *Valsonectria inflata* culture, including the mycelia, was combined with an equal volume of ethyl acetate (50 mL; 1:1, *v*/*v*) and shaken on a rotary shaker at 150 rpm and 28 °C for 3 h. The mixture was subsequently sonicated in an ultrasonic bath for two 15 min intervals. Following centrifugation, the upper ethyl acetate layer was recovered and evaporated to dryness using a rotary evaporator. The obtained extract was then weighed and dissolved in DMSO at appropriate concentrations for subsequent assays.

### 4.3. Coronavirus Infection

HCoV-OC43 was obtained from the American Type Culture Collection (ATCC) (Rockville, MD, USA), and rhabdomyosarcoma (RD) cells were purchased from the Korean Cell Line Bank (Seoul, Republic of Korea). RD cells were maintained in DMEM medium (Welgene, Gyeongsan, Republic of Korea) supplemented with 10% fetal bovine serum (FBS; Thermo Fisher Scientific, Waltham, MA, USA) and 1% penicillin–streptomycin solution (Welgene, Gyeongsan, Republic of Korea).

Coronavirus infection was performed using a previously established protocol [17]. Briefly, RD cells were inoculated with virus-containing medium at an MOI of 0.01 (10^6^ PFU/mL) using the indicated dilutions. After viral infection, the cells were incubated in MEM (Welgene, Gyeongsan, Republic of Korea) supplemented with 2% FBS and 1% penicillin–streptomycin.

For plaque assays, viral supernatant was harvested at 72 h post-infection and passed through 0.45 µm CA membrane filters (Sartorius, Göttingen, Germany). RD cells were seeded in 12-well plates at a density of 4 × 10^4^ cells/mL and infected with the prepared viral supernatants at the indicated concentrations. After 1 h of infection, the cells were overlaid with a mixture containing 0.6% agarose and 2× MEM (Welgene, Gyeongsan, Republic of Korea). Following incubation at 33 °C for 4 days to allow plaque development, the cells were fixed with 4% paraformaldehyde and stained with 0.25% crystal violet solution. Cell viability was evaluated using the MTT assay according to a previously described protocol [35].

### 4.4. Western Blotting

Coronavirus protein expression was analyzed by Western blotting using an anti-HCoV-OC43 antibody. Cell lysates and the viral supernatant were collected separately and prepared using lysis buffer containing 150 mM NaCl, 50 mM HEPES (pH 7.5), and 1% NP-40, supplemented with a protease inhibitor cocktail (Roche, Mannheim, Germany).

Protein concentrations were determined using the Bradford assay, and equal amounts of protein were separated by SDS–PAGE and subsequently transferred to PVDF membranes (Cytiva, Marlborough, MA, USA). The membranes were blocked with 3% skim milk in TBS-T buffer (TBS containing 0.1% Tween-20) and incubated with an anti-HCoV-OC43 primary antibody (MAB-9012, Sigma-Aldrich, St. Louis, MO, USA). An anti-HCoV-OC43 antibody detects the nucleocapsid protein of HCoV-OC43 [20]. Protein bands were detected using the ChemiDoc Imaging System (Bio-Rad, Hercules, CA, USA).

### 4.5. Quantitative RT-PCR

Quantitative RT-PCR analysis was performed to determine coronavirus RNA levels in both cell lysates and the viral supernatant. Cells and culture media were harvested separately, and total RNA was isolated using the PURE™ Total RNA Extraction Kit (Infusion Tech, Anyang, Republic of Korea) according to the manufacturer’s protocol.

Equal amounts of RNA were reverse-transcribed into cDNA using the M-MLV cDNA Synthesis Kit (Enzynomics, Daejeon, Republic of Korea). Real-time PCR amplification was performed using 2× Real-Time PCR Master Mix containing SYBR Green (BioFACT, Daejeon, Republic of Korea) on the QuantStudio 3 Real-Time PCR System(Applied Biosystem, Thermo Fisher Scientific, Waltham, MA, USA). RPL4 gene expression was used for normalization. Primer sets targeting viral and cellular genes were adopted from a previously published study [17].

### 4.6. Scanning Electron Microscopy

For scanning electron microscopy (SEM) analysis, RD cells were seeded onto sterilized 9 mm coverslips and infected with HCoV-OC43 for 72 h. Following infection, the cells were fixed in 2.5% glutaraldehyde for 1 h and sequentially dehydrated using increasing concentrations of ethanol (20%, 40%, 60%, 80%, 90%, and 100%).

The samples were subsequently dried in a vacuum desiccator for 30 min. After platinum coating, cellular morphology was examined using a SUPRA 40 scanning electron microscope (Carl Zeiss, Oberkochen, Germany).

### 4.7. QTOF-MS Analysis of V. inflata Extract

LC-MS analysis was performed using a Waters ACQUITY UPLC I-Class system (Waters Co., Milford, MA, USA) coupled to a Waters Xevo G2 QTOF mass spectrometer (Waters Corp., Manchester, UK) at the Seoul Center of the Korea Basic Science Institute (CC104 and WS 006). Chromatographic separation was achieved on a Waters ACQUITY UPLC BEH C18 column (150 mm × 2.1 mm, 1.7 μm). The autosampler and column oven temperatures were maintained at 10 °C and 40 °C, respectively. The flow rate was set to 300 μL/min, and the injection volume was 2 μL. The mobile phases consisted of 0.1% formic acid in water as solvent A and 0.1% formic acid in acetonitrile as solvent B. Chromatographic separation was performed using gradient elution from 10% to 90% solvent B over 14 min.

Mass spectrometric analysis was performed using an electrospray ionization (ESI) source in positive ion mode. MS/MS fragmentation data were acquired in MSE mode using a collision energy ramp from 25 to 50 eV. The ESI parameters were as follows: capillary voltage, 2.5 kV; cone voltage, 30 V; source temperature, 120 °C; desolvation temperature, 400 °C; cone gas flow, 50 L/h; and desolvation gas flow, 800 L/h. Data were acquired over an m/z range of 80–1200. Instrument calibration was performed using sodium formate as the calibration standard. Leucine enkephalin at m/z 556.2771 in positive ion mode was used as the reference lock mass at a concentration of 200 pg/μL and a flow rate of 5 μL/min. Data acquisition was controlled using MassLynx V4.1 software (Waters Corporation, Milford, MA, USA), and metabolite annotation was performed using UNIFI software, version 1.8.

### 4.8. Statistical Analysis

Data from Western blotting, quantitative RT-PCR, and MTT assays were statistically evaluated by one-way ANOVA followed by Dunnett’s post hoc test using GraphPad Prism software, version 8.0.2 (GraphPad Software, Boston, MA, USA). Differences were considered statistically significant at *p* < 0.05. IC_50_ values were calculated by nonlinear regression analysis using GraphPad Prism. Dose–response curves were fitted using a four-parameter logistic model for inhibition. The IC_50_ values are reported with 95% confidence intervals.

## 5. Conclusions

Fungi are valuable sources of structurally diverse secondary metabolites with a broad range of biological activities, including antimicrobial and antiviral effects. In this study, we demonstrated that extracts from *V. inflata* inhibit human coronavirus OC43 replication in RD cells. VIE treatment reduced coronavirus protein expression, viral RNA levels, and plaque formation, indicating that VIE suppresses viral replication and the production of infectious progeny virus. In addition, SEM analysis showed a reduced number of visible coronavirus particles on the surface of VIE-treated cells, further supporting the antiviral effect of VIE at the morphological level.

Together, these findings provide consistent evidence from molecular, virological, and morphological analyses that *V. inflata* extract has antiviral activity against human coronavirus. Given the continuous emergence of coronavirus variants and the need for novel antiviral resources, our study suggests that *V. inflata* may serve as a promising source of bioactive compounds for the development of antiviral agents. Further studies are required to identify the active antiviral constituents of VIE and to clarify their mechanisms of action.

## Figures and Tables

**Table 1:** IC_50_ values, 95% confidence intervals, and R^2^ values of VIE for the inhibition of HCoV-OC43 replication, as determined by qRT-PCR.

	Gene	IC_50_ (µg/mL)	R^2^	CI
Cell lysate	RdRp	13.55	0.8515	nd–16.98
	M	12.67	0.9081	nd–15.24
	N	13.01	0.9044	nd–15.63
Viral supernatant	RdRp	8.67	0.9030	6.67–10.88
	M	6.47	0.8651	3.55–8.99
	N	8.14	0.9573	7.02–9.36

CI, 95% confidence interval; R^2^, R-square value; nd, not determined.

**Table 2:** Putative annotation of chemical constituents detected in VIE based on UPLC-QTOF-MS analysis in positive ion mode.

RT (min)	Putative Annotation	Molecular Formula	Observed *m*/*z* [M + H]^+^	Mass Error (ppm)	Response	MS/MS Fragments (*m*/*z*)	Refs.
1.60	Gentisyl alcohol	C_7_H_8_O_3_	141.0548	1.2	54,693	-	
2.43	Hydroxytyrosol	C_8_H_10_O_3_	155.0705	1.4	82,016	-	
2.54	Cyclo (Leu-Pro)	C_11_H_18_N_2_O_2_	211.1446	2.1	56,465	98.0595, 138.1278	[22]
5.50	Pyridone alkaloid derivative	C_14_H_19_NO_4_	266.1392	2.0	60,097	-	
6.69	Steroidal alkaloid derivative	C_27_H_43_NO_2_	414.3373	1.5	191,461	-	
6.91	*N*-Benzyl-3-phenylpropanamide	C_16_H_17_NO	240.1388	2.0	213,581	91.0535, 105.0696	
7.67	*N*-Lauryldiethanolamine	C_16_H_35_NO_2_	274.2746	1.8	749,606	88.0750, 106.0859	[23]
7.74	Aminotetradecanol	C_14_H_31_NO	230.2482	1.7	256,418	-	
7.83	Terpenoid derivative	C_27_H_36_O_6_	457.2585	−3.9	328,478	-	
8.15	Sphinganine (d17:0)	C_17_H_37_NO_2_	288.2901	1.2	308,546	252.1203, 270.2791	[23]
8.22	Tris(2,2-dimethylpropionylamino)benzene	C_21_H_33_N_3_O_3_	376.2599	1.0	860,626	-	
8.35	Aminoheptadecanetriol	C_17_H_37_NO_3_	304.2849	1.1	289,693	286.2744	[24]
9.22	Sphinganine (d18:0)	C_18_H_39_NO_2_	302.3062	2.9	455,733	284.2956	[23]
9.67	Sphinganine (d19:0)	C_19_H_41_NO_2_	316.3216	1.8	168,332	298.3124	[23]
9.77	Indole alkaloid derivative	C_28_H_42_N_2_O_2_	439.3326	1.5	182,089	-	
9.82	17-Methyl-phytosphingosine	C_19_H_41_NO_3_	332.3167	2.3	143,317	-	
10.66	Sphinganine (d20:0)	C_20_H_43_NO_2_	330.3373	1.9	236,985	312.3269	[23]
12.48	Dimeric meroterpenoid derivative	C_42_H_56_O_6_	657.4172	3.4	1,237,047	-	
12.52	Triterpenoid derivative	C_39_H_59_NO_8_	670.4301	−1.8	2,652,599	-	
12.82	Triterpenoid derivative	C_43_H_58_O_6_	671.4326	2.9	502,604	-	
12.90	Triterpenoid derivative	C_42_H_56_O_6_	657.4171	3.2	795,071	-	
13.15	Arthrinic acid	C_32_H_54_O_9_	582.3773 [M]^+^	0.9	375,690	-	
13.25	Triterpenoid derivative	C_30_H_52_O_9_	556.3612 [M]^+^	−0.8	181,399	-	
13.42	Triterpenoid derivative	C_30_H_52_O_9_	556.3616 [M]^+^	0.1	441,002	-	
13.65	Triterpenoid derivative	C_31_H_47_NO_6_	530.3482	1.1	915,084	-	
13.78	Diacylglycerol derivative	C_41_H_68_O_5_	641.4225	−2.9	900,181	-	[25]
13.87	Diacylglycerol derivative	C_41_H_68_O_5_	641.4227	−2.3	686,920	-	[25]

## Data Availability

Data are contained within the article.

## Supplementary Materials

The following supporting information can be downloaded at: https://www.mdpi.com/article/10.3390/ijms27146328/s1.
