# Supplementary material for: Extracts from Valsonectria inflata, a Soil-Derived Fungus, Inhibit Human Coronavirus OC43 Replication
Source: Int J Mol Sci. 2026 Jul 16;27(14):6328. doi: 10.3390/ijms27146328 (PMC13410906; doi:10.3390/ijms27146328)
Supplement: Supplementary file 1 [file ijms-27-06328-s001.zip › ijms-4391950-supplementary.pdf]

Characterization of *Valsonectria inflata*

Valsonectria inflata is an acremonium-like fungus that was previously classified as *Gliomastix inflata* and *Acremonium inflatum*. Morphologically, *V. inflata* is characterized by hyaline, septate, and branched hyphae bearing slender phialidic conidiogenous cells. The phialides are mostly lateral or terminal, narrow, and tapered toward the apex, measuring 8–18 μm in length and 1.0–1.5 μm in width. The conidia are hyaline, smooth-walled, unicellular, ellipsoidal to ovoid, and occasionally slightly curved, measuring approximately 2.3–3.8 × 1.4–2.4 μm.

Colonies grown on PDA for 7 days at 25 °C reached 30–34 mm in diameter. The colonies were circular to subcircular, white to off-white on the surface, and cottony to floccose, with abundant aerial mycelium. The colony center was relatively dense and slightly raised, whereas the margin was lobate to undulate. The reverse side was pale cream to pale yellowish, with a slightly darker central region.

Phylogenetic analysis was conducted using 12 nucleotide sequences. Ambiguous positions were removed for each sequence pair using the pairwise deletion option, resulting in a final dataset of 1,280 positions. Evolutionary distances were calculated using the Kimura 2-parameter model and are expressed as the number of base substitutions per site. Rate variation among sites was modeled using a gamma distribution with a shape parameter of 1.00. The optimal tree, with a total branch length of 0.358, is shown in Supplementary Figure 1. The tree is drawn to scale, with branch lengths corresponding to the evolutionary distances used to infer the phylogenetic relationships. Bootstrap values based on 1,000 replicates are indicated next to the branches and represent the percentage of replicate trees in which the associated taxa clustered together.

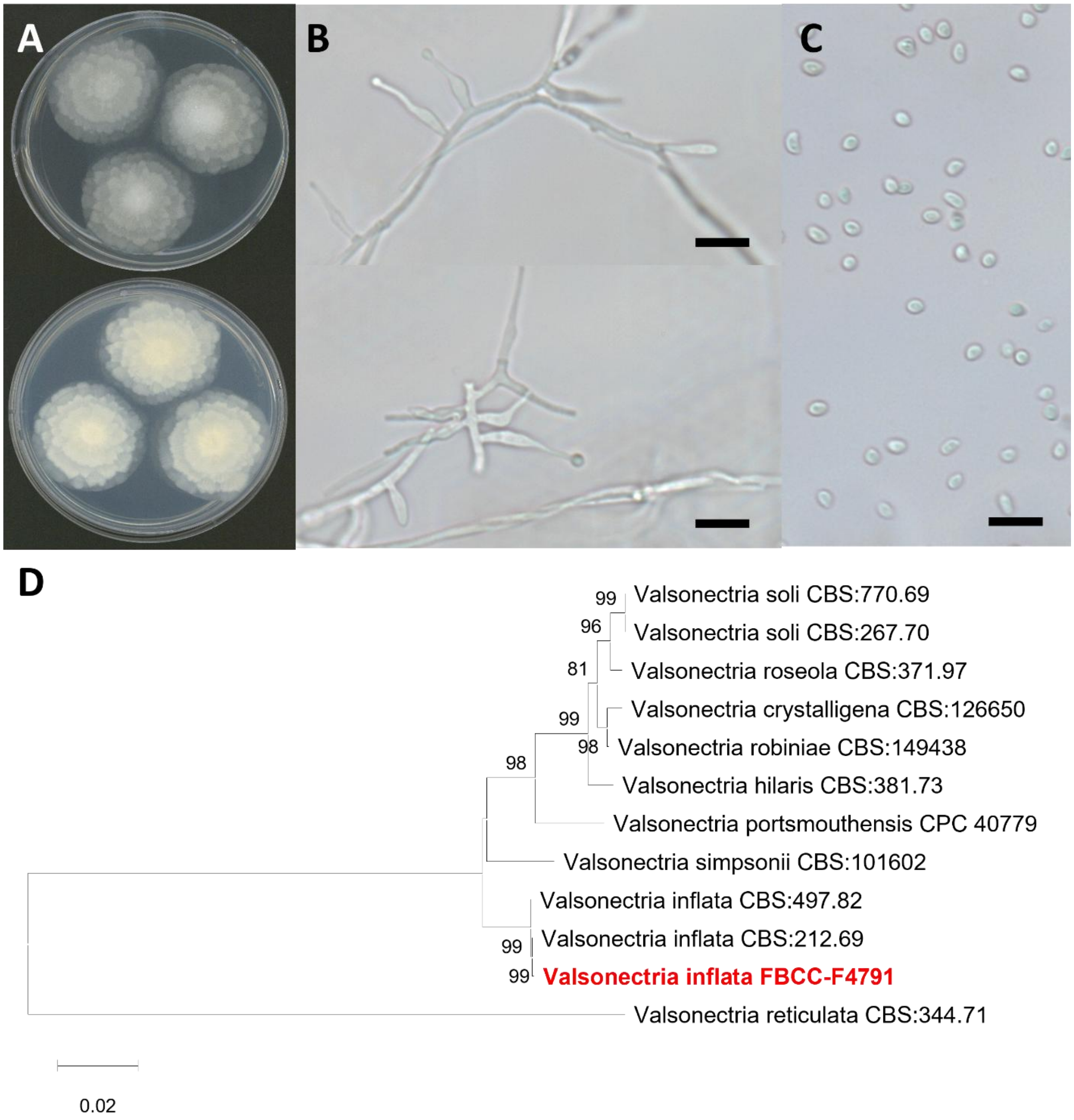

Supplementary Figure S1. Morphological and phylogenetic characteristics of *Valsonectria inflata*. (A) Colony morphology on PDA. (B) Phialidic conidiogenous cells. (C) Conidia. (D) Neighbor-Joining phylogenetic tree based on ITS and LSU sequence data showing the phylogenetic position of *V. inflata* among related taxa. *Valsonectria reticulata* was used as the

outgroup. Scale bars in (B) and (C) = 10 µm. FBCC-F4791 is the deposition number assigned by the Freshwater Bioresources Culture Collection in Sangju, Korea.
